# Carbides Evolution in a Ni-16Mo-7Cr Base Superalloy during Long-Term Thermal Exposure

**DOI:** 10.3390/ma10050521

**Published:** 2017-05-12

**Authors:** Fenfen Han, Li Jiang, Xiangxi Ye, Yanling Lu, Zhijun Li, Xingtai Zhou

**Affiliations:** Center for Thorium Molten Salt Reactor System, Shanghai Institute of Applied Physics, Chinese Academy of Sciences, Shanghai 201800, China; jiangli@sinap.ac.cn (L.J.); yexiangxi@sinap.ac.cn (X.Y.); luyanling@sinap.ac.cn (Y.L.); lizhijun@sinap.ac.cn (Z.L.); zhouxingtai@sinap.ac.cn (X.Z.)

**Keywords:** nickel base superalloy, carbide, thermal exposure, in situ transformation

## Abstract

The effect of long-term thermal exposure on the carbide evolution in a Ni-16Mo-7Cr base superalloy was investigated. The results show that M_12_C carbides are mainly precipitated on the grain boundaries during thermal exposure, and the primary massive M_6_C carbides can be completely transformed to M_12_C carbides in situ at temperatures above 750 °C for long-term thermal exposure. The transformation from M_6_C carbides to M_12_C carbides is attributed to the release of C atoms from M_6_C, which results in the morphology changes of massive carbides, and stabilization of the sizes of M_12_C carbides precipitated on the grain boundaries.

## 1. Introduction

Molten salt reactor (MSR) is considered one of the most promising generation IV nuclear reactors due to its numerous operational and safety advantages [1,2,3]. A wrought Ni-16Mo-7Cr base superalloy named GH3535 is developed and used as the structure material for MSR applications. It is solid-solution strengthened with 16% Mo, and contains 7% Cr for moderate oxidation resistance, which has superior corrosion resistance in molten fluoride salt and good mechanical properties [4,5,6,7]. The microstructure is composed of γ matrix with equiaxial grain size of about 70 μm and stringers of massive primary precipitates, which are determined as M_6_C carbides (M represents the metal elements).

Because this alloy is supposed to be applied at about 700 °C for more than ten years in MSR, the thermal stability and mechanical properties of the alloy are always major concerns for designers. The previous research into the thermal stability of the Ni-16Mo-7Cr base alloy showed that the stringers of primary M_6_C carbides remained stable during the process of solid solution heat treatment [8]. Liu et al. studied the effect of long-term thermal exposure on microstructure and stress rupture properties of GH3535 superalloy, and suggested that the secondary carbide precipitated on the grain boundary is M_12_C, which coexists with primary M_6_C carbides in the aged alloy at 700 °C [7], while Leitnaker et al. mentioned that only M_12_C exists in a Ni-16Mo-7Cr base alloy exposed at 815 °C for 10,000 h [9]. Both studies emphasized that the precipitated carbide was M_12_C rather than M_6_C, but did not mention whether the transformation of primary M_6_C to M_12_C carbides occurs during thermal exposure. Because the carbides play a major role in strengthening the alloy, it is worth evaluating the type and evolution of the carbides in the longer thermal exposure time at elevated temperatures. 

In this study, the evolution of carbides in GH3535 alloy is systematically investigated in the temperature range of 600–800 °C. The types, stability, and evolution of carbides are discussed and determined in this paper.

## 2. Materials and Methods

The nominal chemical composition (wt. %) of the investigated alloy in this study is: Mo, 16; Cr, 7; Fe, 4; Mn, 0.5; Si, 0.5; C, 0.05; Ni, balance. The master alloy was prepared by vacuum induction melt-furnace (VIM), followed by hot forging and rolling into rods with the diameter of 16 mm in the temperature range of 1150–1200 °C. The specimens cut from the rods were solution heat-treated at 1177 °C for 40 min, followed by water quenching, and then placed in the furnaces at the temperatures of 600 °C, 650 °C, 700 °C, 750 °C, and 800 °C. The time intervals for each temperature were 1000 h, 3000 h, 6000 h, and 10,000 h. The thermally exposed specimens were removed from the furnace and cooled in air after thermal exposure. Then, these specimens were ground using metallographic SiC papers to #2000, polished by diamond grinding paste, and chemically etched by a solution of 3 g CuSO_4_ + 10 mL H_2_SO_4_ + 40 mL HCl + 50 mL H_2_O with 20 s for microstructure observation. 

A scanning electron microscope (MERLIN Compact, Carl Zeiss, Oberkochen, Germany) with voltage of 15 kV was employed to observe the microstructure morphology of the specimens. An electron probe microanalyzer (EPMA-1720H, SHIMADZU, Kyoto, Japan) with voltage of 15 kV and current of 50 nA was used to quantitatively analyze the composition of the carbides. An X-ray diffraction (D8 Advance, Bruker, Madison, WI, USA) system with voltage of 40 kV and current of 40 mA was used to identify the phase type in the alloy. The size of specimens used for SEM and EPMA analysis was 10 mm × 10 mm × 5 mm, while the specimen used for XRD analysis was powder prepared by electrochemical extraction method. The exposed alloy as anode was electrolyzed in the solution of CH_3_OH and HCl (3:7) with voltage of 10 V for 24 h. Then, the powder specimen was obtained from the achieved solution by centrifugal machine separation, followed by repeated cleaning by alcohol and air drying for XRD analysis.

## 3. Results

**Figure 1:**
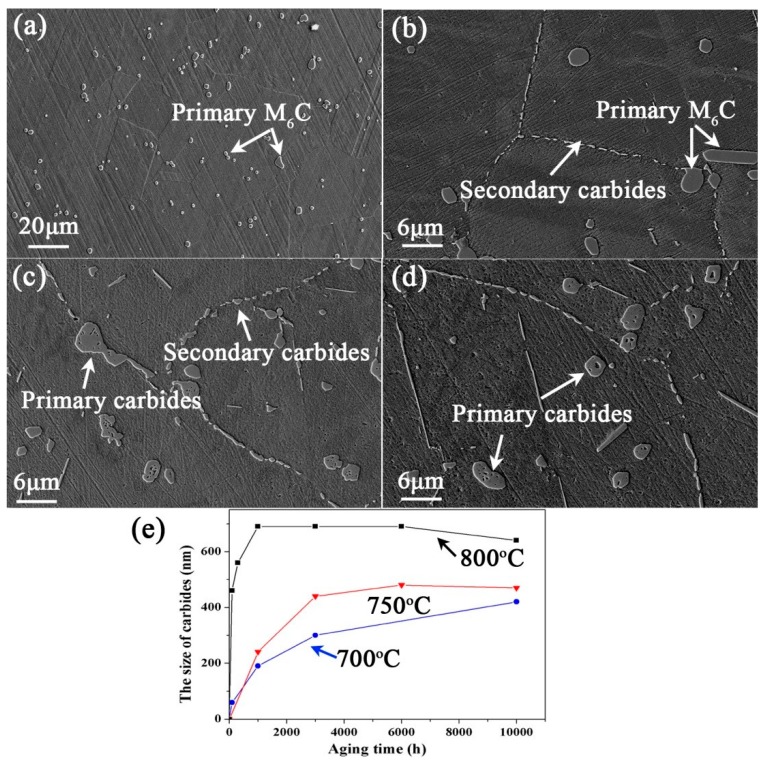
The microstructure of the alloy exposed at 750 °C for various times: (**a**) 0 h (heated alloy); (**b**) 1000 h; (**c**) 3000 h; (**d**) 10,000 h and (**e**) the size of carbides precipitated on the grain boundary. The data at 700 °C in Figure 1e referred to Ref. [10].

The evolution of the microstructures and mechanical properties of GH3535 alloy were investigated at 700 °C [7]. The focus of this research is mainly on the evolution of primary carbides at higher temperatures. Figure 1 shows the microstructure evolution of the experimental alloy exposed to 750 °C for various times. The heat-treated alloy consisted of equiaxed grains and primary M_6_C carbides distributed in the grain or on the grain boundary, as shown in Figure 1a. When the alloy is subjected to thermal exposure, the secondary carbides of M_12_C are precipitated on the grain boundary (as shown in Figure 1b–d), which were also identified in previous work [7]. For the alloy exposed for more than 3000 h, the primary carbides were no longer dense, and lots of holes are observed inside of the massive primary carbides. The sizes of M_12_C carbides in the alloy exposed at 750 °C and 800 °C are shown in Figure 1e. At least 10 different SEM images with 10,000-fold magnification were used to determine the sizes of grain boundary carbides in the specimen with each exposure condition. Here, the size of M_12_C carbide is defined as the average data of the widths of the carbides in the vertical direction of the grain boundaries. It can be seen that the size of M_12_C carbide reached 450 nm after the alloy was exposed at 750 °C for 3000 h and it no longer increased afterwards. As the alloy was exposed at 800 °C, the size of M_12_C no longer increased after 1000 h and the maximal size was about 700 nm. However, for the alloy exposed at 700 °C, the size of grain boundary carbide kept increasing with the rising of the exposure time (the data at 700 °C in Figure 1e refer to Ref. [10]).

**Figure 2:**
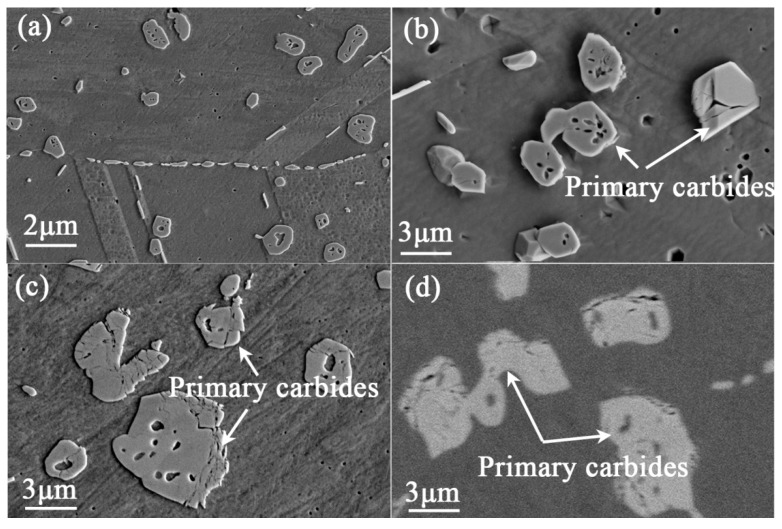
SEM images for the morphology of primary carbides in the alloy exposed at 800 °C with chemical etching: (**a**,**b**) 1000 h; (**c**) 3000 h and without etching (**d**) 3000 h.

Figure 2 shows the morphologies of the primary carbides during the exposure process at 800 °C for various exposure times. After the alloy was exposed for 1000 h, holes and cracks were apparent in the interior of the primary carbides, as shown in Figure 2a,b. With the increasing of the exposure time, more cracks were generated and the sizes of the holes obviously increased in the massive primary carbides (Figure 2c). The specimen without chemical etching is also presented in Figure 2d, and the holes and cracks can be observed clearly in the primary carbides. It is proved that the formation of these holes is caused by the thermal exposure. 

**Figure 3:**
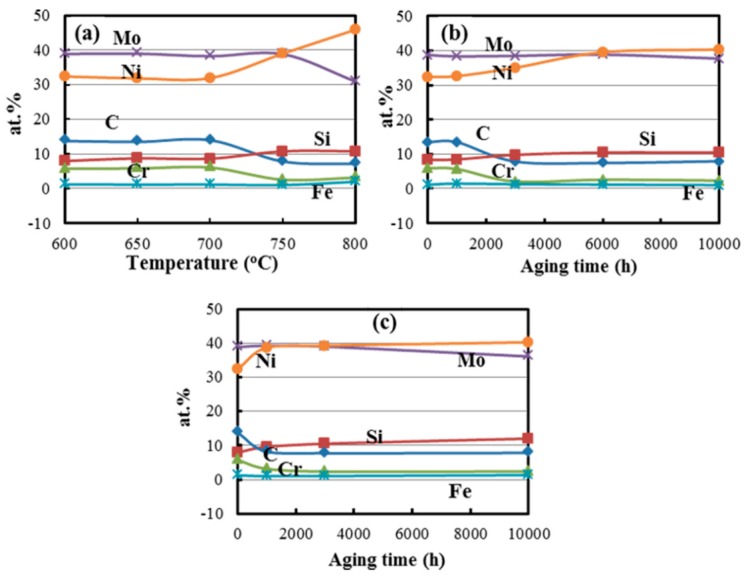
The elements evolution in massive primary carbides: (**a**) The alloy exposed for 10,000 h at different temperature; The alloy exposed at (**b**) 750 °C and (**c**) 800 °C for 10,000 h.

The change in morphology of massive primary carbides may indicate the change in the composition, and thus EPMA quantitative analysis was also carried out for the primary carbides in the alloy exposed at various conditions, as shown in Figure 3. The data for each condition are the average data of at least ten primary carbides in the specimen. When the alloy is exposed at different temperatures for 10,000 h, the C and Cr contents of the M_6_C carbides decrease by half at temperature above 750 °C, while the Si and Ni contents of those increase (Figure 3a). For the alloy exposed at 750 °C and 800 °C, the phenomenon of the composition change occurs after 3000 h and 1000 h, respectively (Figure 3b,c). 

**Figure 4:**
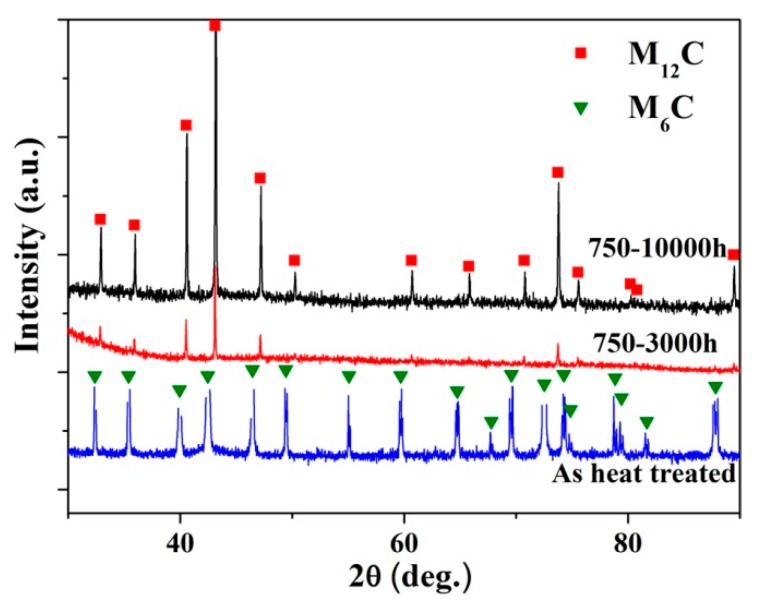
X-ray diffraction pattern of the specimen aged at 750 °C for various times.

In order to determine whether there is any type change of the primary carbides with the composition change in Figure 3, XRD analyses were employed on the powder of carbides obtained from the exposed alloys. Figure 4 shows X-ray diffraction pattern of the specimen aged at 750 °C for various exposure times. For the heat-treated alloy, only M_6_C was detected in the carbides powder obtained by chemical extraction from the alloy, while only one type of carbide, M_12_C, was detected for the alloy exposed longer than 3000 h and 10,000 h. This suggests that the primary M_6_C carbides transform to M_12_C carbides in the thermal exposure process. According to the microstructure observation in Figure 1 and composition analyses in Figure 3, the transformation is in situ just through composition change and lattice reconfiguration of M_6_C carbides.

## 4. Discussion

The original microstructure of heat-treated GH3535 alloy was characterized by stringers of massive primary M_6_C carbides. When the alloy is subjected to thermal exposure, M_12_C carbides are precipitated on the grain boundaries [7,11]. When the alloy was exposed to temperatures above 750 °C for a long time, the massive M_6_C carbides were transformed to M_12_C carbides (Figure 3 and Figure 4). The findings in this work are different from the previous studies for the GH3535 alloy exposed at 700 °C with long-term thermal exposure, where two types of carbides coexist in the exposed alloy [11]. 

The crystal structures of the M_12_C and M_6_C carbides were almost identical, both of which are in fcc crystal structure. The major difference is that the number of carbon atoms in M_12_C carbide is half that in M_6_C in the unit cell, and the lattice parameter of the M_12_C carbide is a little smaller than that of M_6_C (M_12_C, 1.089 nm; M_6_C, 1.105 nm) [12,13]. Thus, the literature about the transformation from M_6_C to M_12_C is relatively scarce in nickel base superalloys. For the GH3535 alloy, in the matrix, the contents of alloying elements that form carbides gradually decrease due to the precipitation of M_12_C carbides on the grain boundaries, which can break the concentration balance of alloying elements in the alloy, and then result in the escape of C atoms from M_6_C carbides into the matrix (Figure 3). Consequently, the M_6_C carbides completely transform to M_12_C carbides by the transport of C atoms into the matrix due to the structural similarity of two kinds of carbides. This transformation has also been observed in cobalt-based alloys [14,15]. The large release of C atoms from M_6_C carbides into matrix increases the velocity of nucleation and growth of secondary carbides precipitated on the grain boundaries, and thus increases their size until a new balance of C concentration is achieved in the aged alloy. On the other hand, the obvious diffusion of C, Si, and Cr atoms across the phase interface between carbides and the matrix exists during the long-term thermal exposure (Figure 3), and the lattice parameter decreases in the transformation from M_6_C carbides to M_12_C carbides. We simplified the M_6_C and M_12_C carbides as Ni_3_Mo_3_C and Ni_6_Mo_6_C structures, respectively, and calculated their densities as 9.031 g/cm^3^ and 9.517 g/cm^3^ by dividing the atomic mass by volume in the unit cell [12,16]. Provided that the phase interface does not migrate, the change of the density of carbide particles will cause the so-called Kirkendall pores (Figure 2). Such a difference in the density can cause five pores with a diameter of 1μm in the spherical particle with a diameter of 10 μm, which is in good accordance with the morphologies shown in Figure 2c.

## 5. Conclusions

The effect of long-term thermal exposure on carbides evolution was systematically investigated in a Ni-16Mo-7Cr base alloy, and the main conclusions were as follows:

1. The secondary M_12_C carbides are mainly precipitated on the grain boundaries during thermal exposure, and their sizes reach stable state when the alloy is exposed at 750 °C and 800 °C for 3000 h and 1000 h, respectively.
2. The primary massive M_6_C can be completely transformed to M_12_C in situ at higher temperatures above 750 °C through composition change and lattice reconfiguration.
3. The transformation from M_6_C carbides to M_12_C carbides is attributed to the release of C atoms from M_6_C, which results in the morphology changes of massive carbides, and stabilization of the sizes of M_12_C carbides precipitated on the grain boundaries.
